# Eu^3+^-doped ZnO quantum dots: structure, vibration characteristics, optical properties, and energy transfer process

**DOI:** 10.1039/d4na00858h

**Published:** 2024-12-12

**Authors:** T. T. T. Huong, N. T. Sa, N. T. M. Thuy, P. V. Hao, N. H. Thao, N. T. Hien, N. X. Ca

**Affiliations:** a Ha Noi University of Industry Ha Noi Vietnam huongttt@haui.edu.vn; b Faculty of Physics, TNU-University of Education Thai Nguyen Vietnam; c Faculty of Physics, Ha Noi Pedagogical University 2 Vinh Phuc Vietnam; d Institute of Science and Technology, TNU-University of Sciences Thai Nguyen Vietnam canx@tnus.edu.vn

## Abstract

This article studies the synthesis, as well as the structural, vibrational, and optical properties of Eu^3+^-doped ZnO quantum dots (QDs) and investigates the energy transfer mechanism from the ZnO host to Eu^3+^ ions using Reisfeld's approximation. Eu^3+^-doped ZnO QDs at varying concentrations (0–7%) were successfully prepared using a wet chemical method. The successful doping of Eu^3+^ ions into the ZnO host lattice, as well as the composition and valence states of the elements present in the sample, were confirmed through X-ray diffraction (XRD) and X-ray photoelectron spectroscopy (XPS) analyses. XRD results demonstrated the crystalline nature of the ZnO QDs, revealing their wurtzite (WZ) structure with no secondary phases. XPS analysis provided further confirmation of the presence of Eu^3+^ ions within the ZnO host, with clear signals corresponding to the Zn, O, and Eu elements. The valence states of Eu were verified as trivalent (Eu^3+^), confirming the successful doping of Eu^3+^ ions, as evidenced by the characteristic Eu 3d peaks in the XPS spectra. Raman spectroscopy (RS) was employed to analyze the vibrational modes, revealing shifts in ZnO lattice vibrations due to Eu^3+^ incorporation, indicating strong coupling between Eu^3+^ ions and the ZnO host. Optical properties were studied using UV-Vis absorption, photoluminescence (PL) spectroscopy, and PL decay spectroscopy, showing a significant enhancement of red emission, attributed to the ^5^D_0_ → ^7^F_2_ transition of Eu^3+^ ions under UV excitation. Using Judd–Ofelt (JO) analysis, the intensity parameters (*Ω*_2_, *Ω*_4_, *Ω*_6_) were derived, providing insights into the asymmetry of the Eu^3+^ ion's local environment and the radiative transition probabilities. Energy transfer processes between the ZnO host and Eu^3+^ dopants were examined, showing efficient sensitization of Eu^3+^ through excitation of the ZnO host, with an optimal Eu^3+^ doping level maximizing luminescence. Eu^3+^-doped ZnO QDs, which emit in the visible light region and are non-toxic, have great potential for applications in photonic devices, light-emitting diodes, and bioimaging.

## Abstract 2

Schematic energy level diagram and the energy transfer process from ZnO host to Eu ions.

## Graphical abstract

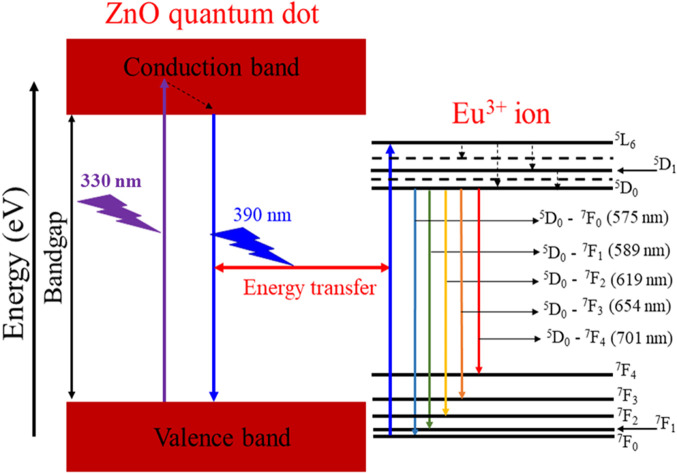

## 1. Introduction

Semiconductor quantum dots (QDs) are nanoscale particles that exhibit unique optical and electronic properties due to quantum confinement effects, where the motion of charge carriers (electrons and holes) is restricted in all three spatial dimensions. This confinement leads to discrete energy levels, making QDs behave similarly to artificial atoms with tunable band gaps.^1–3^ Their size can be precisely controlled, allowing for the customization of their emission wavelength across a broad spectrum, from infrared to ultraviolet. Due to their remarkable photoluminescence and ability to absorb and emit light efficiently, QDs are widely used in applications such as quantum computing, biological imaging, and light-emitting diodes.^3–5^

Zinc oxide (ZnO) QDs have attracted much research interest due to their unique optical, electronic, and chemical properties. ZnO QDs exhibit a wide bandgap (approximately 3.37 eV) and large exciton binding energy (around 60 meV), which make them highly efficient in ultraviolet (UV) light absorption and emission.^6–8^ They have been widely studied for use in UV lasers, light-emitting diodes, and photodetectors.^4,5,7^ Additionally, their good biocompatibility and low toxicity compared to other QDs, such as cadmium-based materials, make ZnO QDs particularly suitable for biomedical applications, including biosensing, bioimaging, and drug delivery.^9–12^ ZnO QDs also possess strong photocatalytic activity, which has been leveraged in environmental applications, such as water purification and pollutant degradation. The synthesis of ZnO QDs is relatively simple, allowing for control of their size, shape, and surface characteristics to optimize their performance in specific applications. Furthermore, ZnO QDs have shown promise in enhancing the performance of solar cells by improving light absorption and charge separation efficiency.^6,8,10^ Their stability, versatility, and wide range of applications make ZnO QDs an important material in nanotechnology research and innovation.^7,11,12^

Trivalent rare earth Eu^3+^ ions have been studied in many areas of materials science due to their unique luminescent properties.^13,14^ These ions are known for their sharp, intense emissions, particularly in the red region of the visible spectrum, which arises from 4f–4f transitions, specifically the ^5^D_0_ → ^7^F_2_ transition. The ability of Eu^3+^ to emit red light with high quantum efficiency has made it a popular dopant in various materials, such as phosphors for lighting and display technologies.^15–17^ More recently, there has been growing interest in incorporating Eu^3+^ ions into semiconductor QDs. The incorporation of Eu^3+^ ions into semiconductor QDs, such as ZnS, ZnSe, CdSe, and CdS QDs, is an exciting area of research because it allows for the creation of new luminescent materials that take advantage of both the quantum confinement effect and the distinctive emission of Eu^3+^.^1,13,14^ These doped QDs exhibit enhanced photoluminescence stability, high color purity, and the potential for multi-functional applications, such as in energy-efficient lighting, displays, and medical diagnostics.^13–16^ The doping of Eu^3+^ into ZnO QDs has attracted significant attention due to their unique structural, vibrational, and optical properties. Eu^3+^-doped ZnO QDs exhibit tunable emission from blue to red by simply changing the excitation wavelength.^15–17^ A broad blue emission from the ZnO host at around 400 nm is observed, while the characteristic red emission from Eu^3+^ ions (^5^D_0_ → ^7^F_2_ transition) becomes dominant under 390–395 nm excitation. This allows for the creation of different color emissions using the same material.^15^ For the first time, Eu-doped ZnO QDs with dual fluorescence emission are reported to be applied in luminescent solar concentrators. The obtained optical efficiency of the luminescent solar concentrators based on Eu-doped ZnO QDs is relatively high because of their high photoluminescence intensity and dual fluorescence emission. Eu^3+^-doped ZnO QDs demonstrate strong solid-state fluorescence, enabling high loading concentrations in polymer films without significant aggregation-induced quenching. This is crucial for applications like luminescent solar concentrators, where high QD loading is necessary to achieve high optical efficiency.^16^ Studies indicated that doping ZnO with Eu^3+^ enhanced its photocatalytic performance compared to that of pure ZnO.^16,17^

Although several studies investigated the doping of Eu^3+^ ions into the ZnO host material, there has been no study on the mechanism and nature of the energy transfer process from the ZnO host to Eu^3+^ ions as well as using JO theory to study the emission characteristics of Eu^3+^ ions in the ZnO host. In this study, we analyze in detail the structural, vibrational, and optical properties of Eu^3+^-doped ZnO QDs using experimental measurements and theoretical models. The Judd–Ofelt theory was employed to analyze the optical transitions and energy transfer dynamics between ZnO and Eu^3+^ ions. This approach will offer insights into the efficiency of energy transfer and the influence of the ZnO host on Eu^3+^ luminescence. The study of these processes not only clarifies the physical mechanisms but also further develops and optimizes the optical properties of Eu^3+^-doped ZnO materials, for their application in optoelectronic devices, bio-imaging, and energy-saving lighting.

## 2. Experimental

### 2.1. Materials

ZAT (Zinc acetate dihydrate-Zn(CH_3_COO)_2_·2H_2_O, 99.99%), OLA (oleylamine –C_18_H_37_N, 97%), ODE (1-octadecene-CH_3_(CH_2_)_15_CH=CH_2_, 95%), TOP (tri-*n*-octylphosphine-C_24_H_51_OP 97%), EuAT (europium acetate hydrate-(CH_3_CO_2_)_3_Eu·*x*H_2_O, 99.9%), isopropanol (70%), and toluene (99.8%) were purchased from Sigma-Aldrich.

### 2.2. Synthesis of Eu^3+^ doped ZnO quantum dots

Eu^3+^-doped ZnO QDs were synthesized *via* a wet chemical method. The synthesis process is based on previous studies.^1,2^ Specifically, 0.03 mol of zinc acetate (ZAT) and europium acetate (EuAT), with the Eu^3+^ concentration determined by the molar ratio of Eu^3+^ to Zn^2+^, were combined with 0.015 mol of trioctylphosphine (TOP) and 50 mL of octadecene (ODE) in a three-neck flask. Argon gas (Ar) was continuously introduced into the system to eliminate residual air. Subsequently, 0.015 mol of oleylamine (OLA) was injected into the reaction flask. The reaction mixture was heated to 280 °C and maintained at this temperature for 60 minutes to achieve monodisperse Eu^3+^-doped ZnO QDs. The resulting QDs were purified for further characterization and analysis. The synthesized samples of ZnO, ZnO : Eu0.5%, ZnO : Eu1%, ZnO : Eu3%, ZnO : Eu5%, and ZnO : Eu7% are denoted as Z0, Z0.5, Z1, Z3, Z5, and Z7, respectively.

### 2.3. Characterization

The structure of QDs was investigated using X-ray diffraction (XRD). The measurements were conducted on a SIMEMS D5005 X-ray diffractometer (Bruker, Germany) with Cu-Kα radiation (*λ* = 1.54056 Å). X-ray photoelectron spectroscopy (XPS) analysis was performed on a Thermo VG Escalab 250 photoelectron spectrometer. RS spectra of the dried samples were measured with a LABRAM-HR800 spectrometer (Jobin Yvon) working with wavelength *λ* = 488 nm (2.54 eV). Ultraviolet-visible (UV-Vis) absorption spectra of the QDs were recorded using a Jasco V-770 spectrometer (Varian). The morphology and size of the QDs were examined *via* transmission electron microscopy (TEM), operated at 100 kV voltage. Photoluminescence (PL), photoluminescence excitation (PLE) spectra, and decay time curves were measured using an FLS1000 spectrophotometric system equipped with a 450 W Xe lamp. All measurements were carried out at room temperature.

## 3. Results and discussion

### 3.1. Structural analysis and vibration characteristics

**Fig. 1:**
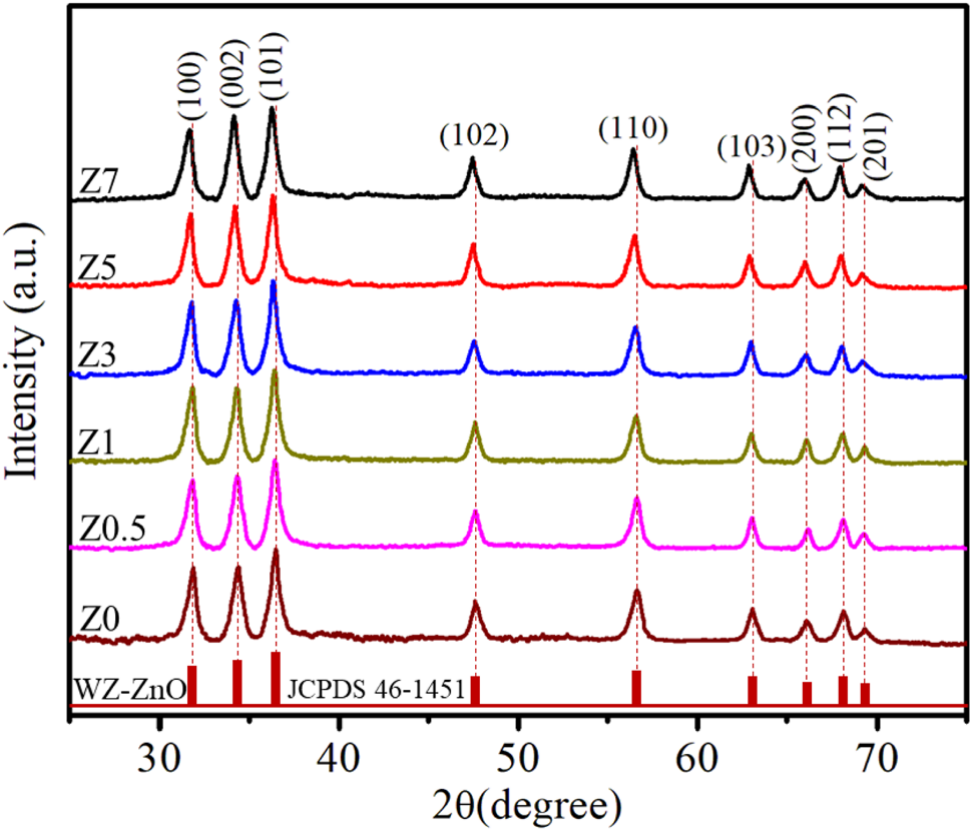
XRD patterns of the Z0, Z0.5, Z1, Z3, Z5 and Z7 samples.

**Fig. 2:**
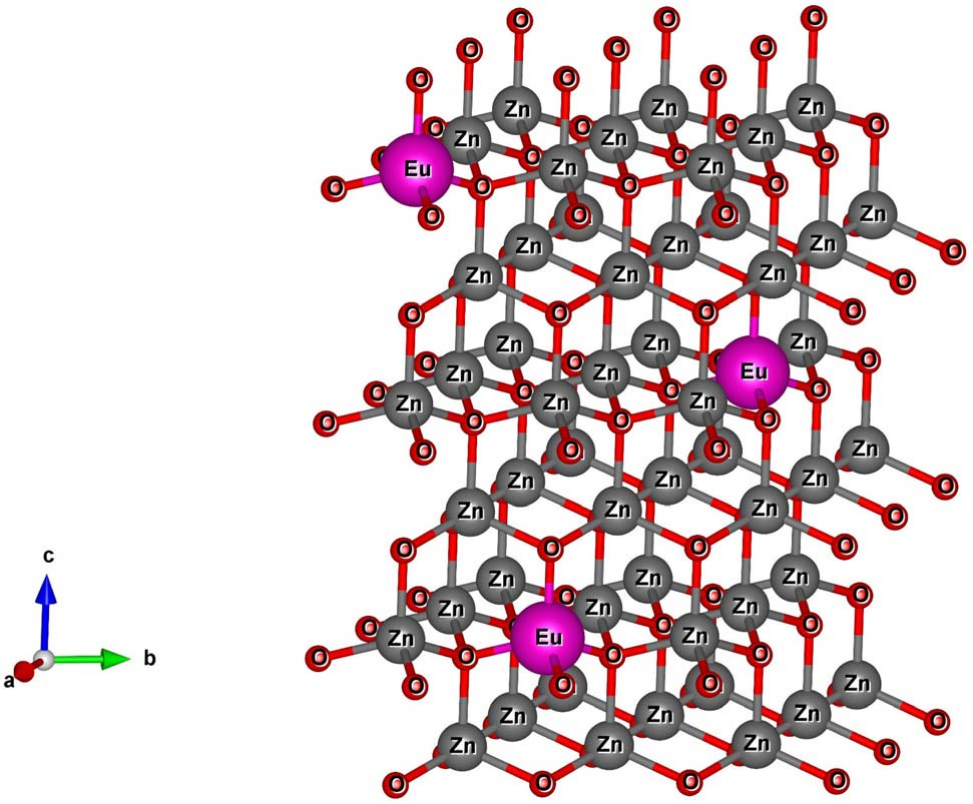
The unit-cell scheme of Eu-doped ZnO QDs with wurtzite structure.

The X-ray diffraction (XRD) patterns of Z0, Z0.5, Z1, Z3, Z5, and Z7 samples are shown in Fig. 1. Diffraction peaks were observed at the (100), (002), (101), (102), (110), (103), (200), (112) and (201) lattice planes. This confirms a wurtzite structure (space group *P*6_3_*mc*, no. 186) of all ZnO and Eu-doped ZnO QDs. All the peak positions of the crystal structure matched well with a hexagonal lattice (ICSD card no. 29272).^18^ There are no discernible impurity peaks in these results, confirming the phase purity of the samples. The unit cell structure of Eu^3+^-doped ZnO with a hexagonal configuration is illustrated in Fig. 2.

Hexagonal structures are typical of ZnO QDs synthesized *via* the wet chemical method.^1,16,18^ A slight shift towards lower angles is observed in the diffraction peaks of Eu^3+^-doped samples (Z0.5, Z1, Z3, Z5, and Z7) compared to pure Z0 (ZnO). This shift is attributed to the substitution of smaller Zn^2+^ ions (radius 0.74 Å) by larger Eu^3+^ ions (radius 0.95 Å under 6-coordination), leading to an increase in the lattice constants.^2,19^ This substitution strains the ZnO lattice, causing a small expansion and a corresponding shift in the diffraction peaks to lower angles (due to the larger atomic spacing). The substitution of Zn^2+^ by Eu^3+^ ions affects the nucleation and growth rate of the QDs, as the larger Eu^3+^ ions induce lattice strain. The gradual shift of the peaks towards lower angles as the concentration of Eu^3+^ increases confirms the increasing replacement of Zn^2+^ ions by Eu^3+^. The crystallite size (*D*) of the QD sample was determined from the broadening of the strongest peak (101) in the XRD pattern using the Debye–Scherrer equation:^1,2,20^1
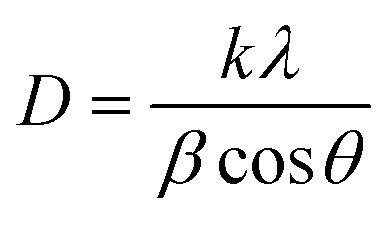
Here, λ is the X-ray wavelength, β is the full width of the diffraction line at half its maximum intensity, and θ is the Bragg angle.

For a hexagonal wurtzite structure, the lattice constants *a* and *c* can be calculated using Bragg's law. The lattice constants are related to the Miller indices (*h*, *k*, *l*) by the following formula:^1^2
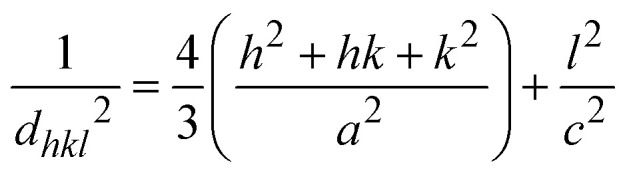
where *d*_*hkl*_ is calculated from Bragg's equation:

3 *nλ* = 2*d*_*hkl*_ sin *θ*

The cell volume (*V*) of a hexagonal unit cell can be calculated using the lattice constants *a* and *c* with the following formula:^1^4
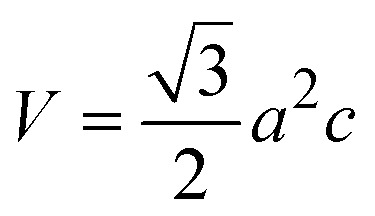


The crystallite strain (*ε*) in the ZnO QDs can be estimated from the peak broadening using the Williamson–Hall equation:^1,2^5
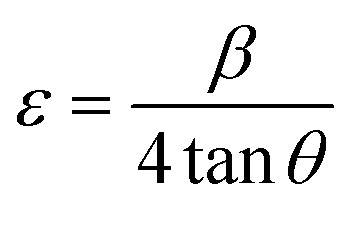


The parameters calculated from the XRD data, including *a*, *c*, 2*θ*, *β*, *V*, *D*, and *ε*, are presented in Table 1. As the concentration of Eu^3+^ increased, the cell volume also increased, which was attributed to the larger ionic radius of Eu^3+^ compared to Zn^2+^. The Eu^3+^ ions occupied the lattice sites of Zn^2+^, leading to an increase in the interatomic distance.^21–23^ The results in Table 1 show that the lattice strain and the crystallite size of Eu^3+^-doped ZnO QDs increased with increasing Eu^3+^ concentration. Chemingui and colleagues proposed that higher Eu^3+^ concentrations cause an excess of Eu^3+^ ions, leading to the formation of Eu–O–Zn bonds on the ZnO surface.^24^ These bonds enhance crystal growth and transfer stress from the outer surface to the interior of the crystal.

**Table 1:** Lattice parameters and crystallite size of Eu^3+^-doped ZnO QDs

Sample	*a* (Å)	*c* (Å)	2*θ* (101)	β × 10^−2^ (rad)	Cell volume (Å^3^)	*D* (nm)	*ε* × 10^−3^
Z0	3.247	5.260	36.52°	1.85	46.80	7.89	1.40
Z0.5	3.249	5.263	36.47°	1.85	46.87	7.89	1.40
Z1	3.251	5.267	36.40°	1.84	46.98	7.93	1.39
Z3	3.253	5.270	36.34°	1.82	47.06	8.02	1.38
Z5	3.255	5.273	36.27°	1.81	47.15	8.06	1.38
Z7	3.257	5.277	36.21°	1.79	47.25	8.15	1.37

**Fig. 3:**
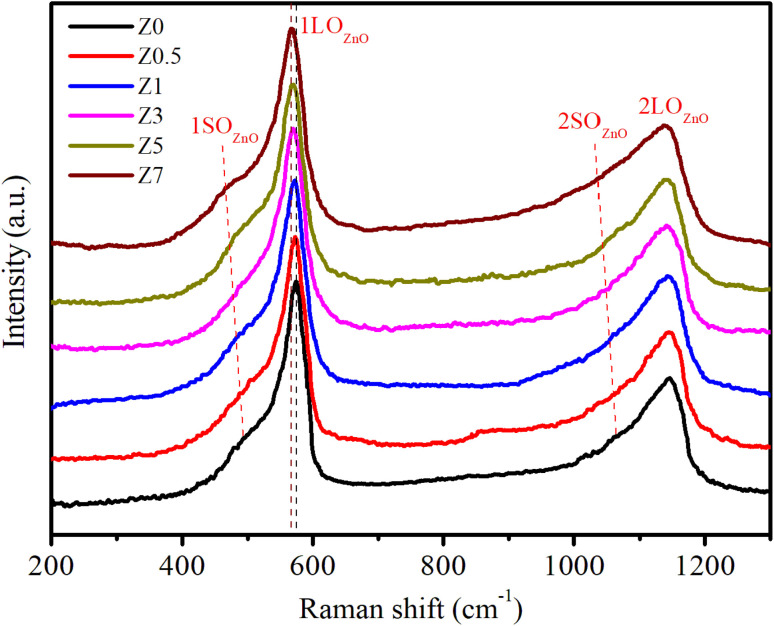
Raman spectra of Eu^3+^-doped ZnO QDs.

Raman scattering (RS) is a powerful tool to investigate the vibrational properties of semiconductors and to explore the effects of doping on the structural properties of materials.^33^Fig. 3 displays the RS spectra of ZnO and Eu^3+^-doped ZnO QDs. For ZnO QDs, the Raman peaks at approximately 573.11 cm^−1^ and 1146.56 cm^−1^ correspond to the first-order longitudinal optical (1LO) phonon mode and its second-order overtone (2LO).^34,35^ The 1LO mode of ZnO QDs is slightly redshifted compared to the 1LO mode in bulk ZnO (which is typically closer to 590 cm^−1^).^34,35^ The redshift of the Raman peak position in ZnO QDs is due to the quantum confinement effect of the nm-sized particles. The spatial confinement of phonons in QDs results in a slight red-shift and peak broadening in the Raman spectrum. In the spatial limit of optical phonons, the Raman spectra of semiconductor QDs exhibit red-shifts and peak broadening due to a relaxation of the *k*-vector selection rule within finite-sized QDs.^36^ According to the Heisenberg uncertainty principle, the fundamental Raman selection rule is relaxed in structures of finite size, allowing phonons located away from the center of the Brillouin zone to participate. The uncertainty in the phonon wavevector scales approximately as 1/*L*, where *L* is the diameter of the QD. This spatial confinement in small-diameter QDs results in red-shifts and asymmetric broadening of Raman peaks in QDs compared to their bulk material.

The 1SO_ZnO_ (first-order surface optical) phonon mode at around 493.34 cm^−1^ is observed in the Raman spectra of ZnO QDs.^33,35^ This mode is attributed to vibrations that are strongly affected by surface states. SO phonon modes in semiconductor QDs are not present in bulk materials. They arise from the vibrations of atoms at or near the surface of QDs. In ZnO QDs, the 1SO mode appears as a distinct feature around 493.34 cm^−1^, which is much lower in energy than the 1LO phonon modes. These surface optical phonons occur due to the strong influence of surface atoms and their interactions with the surrounding environment.^33–35^

For Eu-doped ZnO QDs, when the Eu^3+^ concentration increased from 0.5–7%, the 1LO and 1SO peaks red-shifted from 571.35 to 566.89 cm^−1^ and 486.89 to 466.93 cm^−1^, respectively. The magnitude of this red-shift increases with higher concentrations of Eu^3+^ dopant. Several mechanisms contribute to this red-shift: (i) the ionic radius of Eu^3+^ is larger than that of Zn^2+^, which means that substituting Eu^3+^ for Zn^2+^ introduces strain into the ZnO crystal lattice. This strain weakens the bonds between atoms, reducing the restoring force for atomic vibrations and causing the frequency of the 1LO phonon mode to decrease (red-shift). (ii) Doping with Eu^3+^ can introduce additional defect states, such as oxygen vacancies or interstitials. These defects disrupt the periodicity of the crystal lattice, leading to localized distortions. The increased defect density alters the lattice dynamics and softens the vibrational modes, resulting in a red-shift of the 1LO peak. (iii) In ZnO QDs, the spatial confinement of phonons due to their small size already leads to some red shifting and broadening of the 1LO mode. Increasing the Eu^3+^ ion concentration further enhances this effect by introducing additional stress and disorder, which increases the phonon-limiting effect, resulting in a stronger red-shift.

### 3.2. Elemental composition analysis

**Fig. 4:**
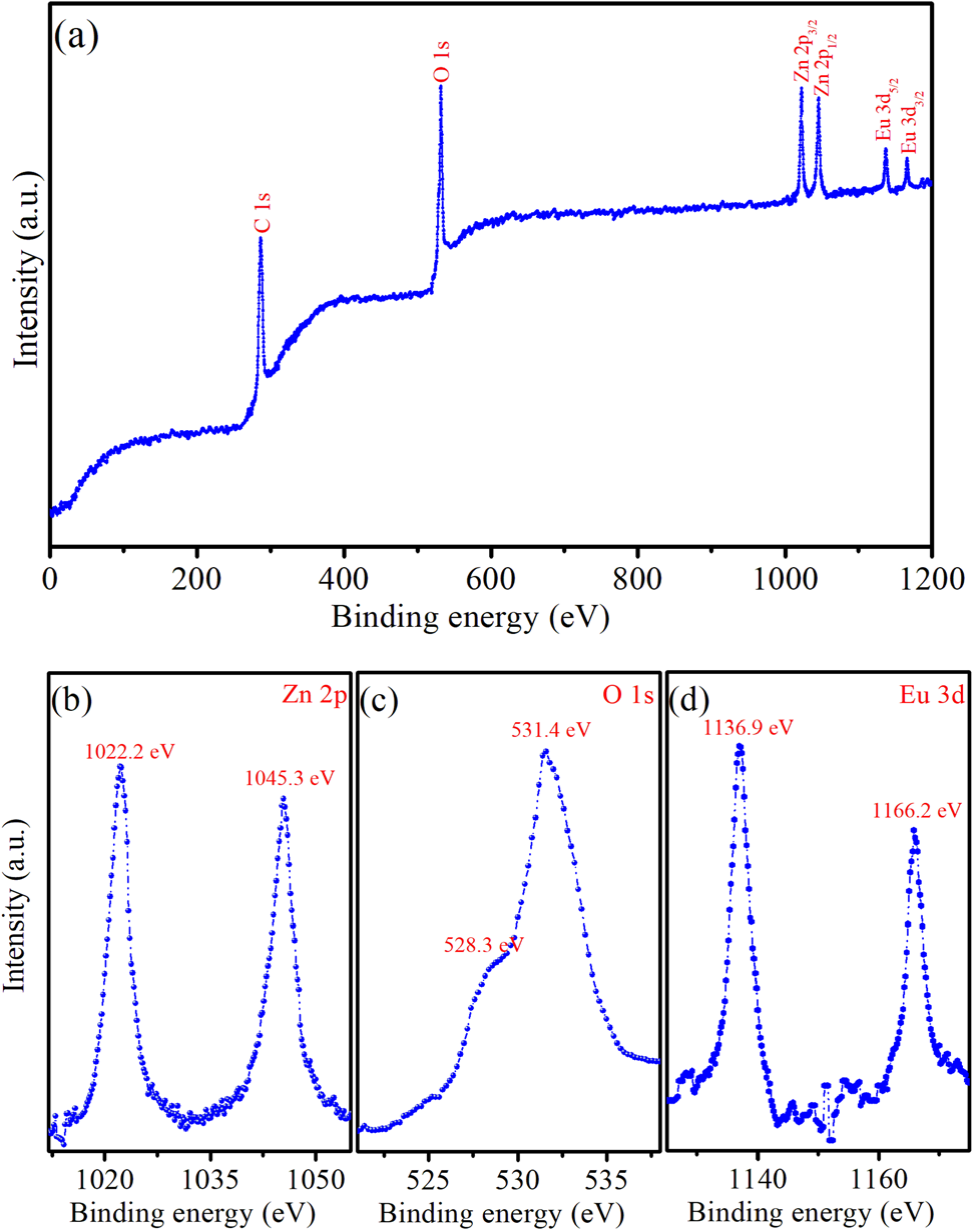
(a) Survey XPS spectrum of Z7 sample, (b) Zn 2p, (c) O 1s, and (d) Eu 3d.

X-ray photoelectron spectroscopy (XPS) provides important information about the chemical state and elemental composition of materials, serving as a key tool to confirm the successful incorporation of impurity elements into the host material. Fig. 4a displays typical XPS survey scans of the ZnO : Eu^3+^7% QDs. This XPS spectrum provides strong evidence of the successful doping of Eu^3+^ ions into ZnO QDs. The Zn-2p, O-1s, and Eu-3d peaks confirm the presence of both the ZnO host lattice and the dopant (Eu^3+^). The XPS survey scan of the ZnO : Eu^3+^7% QDs showed six peaks corresponding to the levels of C-1s, O-1s, Zn-2p_3/2_, Zn-2p_1/2_, Eu-3d_5/2_, and Eu-3d_3/2_. The detection of the C element suggests the presence of residual precursors used during the fabrication process of the QDs. Fig. 4b presents a high-resolution XPS spectrum, where the two distinct peaks at 1022.2 eV and 1045.3 eV are attributed to the Zn-2p_3_/_2_ and Zn-2p_1_/_2_ levels of the Zn element, respectively.^1,2^ The binding energy difference between these peaks is 23.1 eV, which is consistent with previously reported values in the literature.^1,2,25^ The doublet (with 2p_3/2_ and 2p_1/2_) is characteristic of Zn^2+^ in the ZnO lattice, confirming the presence of Zinc in the +2 oxidation state. The strong intensity of the O-1s peak corresponds to oxygen present in the ZnO lattice.^1,25^ This strong intensity peak is actually two very close peaks at 531.4 and 528.3 eV (Fig. 4c). This peak indicates the presence of O^2−^ ions, which are part of the ZnO crystal structure. In ZnO : Eu^3+^ QDs, this peak may also have contributions from Eu–O bonds, particularly at higher concentrations of Eu^3+^. Typical peaks corresponding to Zn-2p and O-1s are evident, representing the characteristic ZnO QDs. The peaks at 1136.9 eV and 1166.2 eV are attributed to Eu-3d_5/2_ and Eu-3d_3/2_ levels, respectively (Fig. 4d).^2,26^ The positions of the Eu-3d_5/2_ and Eu-3d_3/2_ peaks indicate that Eu is present in its trivalent oxidation state (Eu^3+^). The presence of Eu in the spectrum confirms the successful doping of the ZnO QDs with Eu^3+^ ions.

### 3.3. Absorption properties

**Fig. 5:**
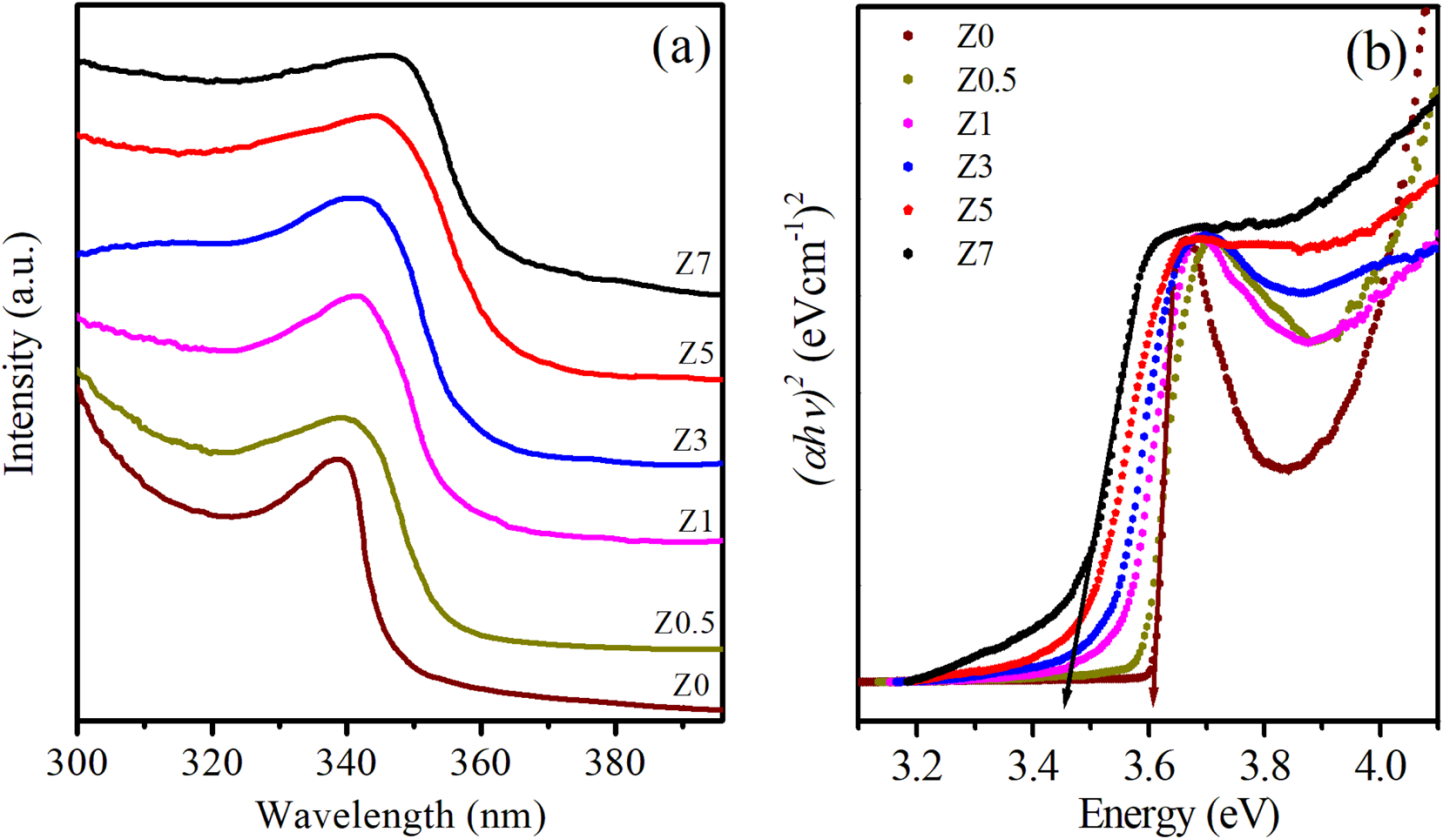
(a) UV-Vis spectra and (b) the plot of variation of (*αhv*)^2^*versus* energy (*hv*) of Eu^3+^-doped ZnO QDs.

The absorption spectra provide valuable information about the optical properties and energy band structures of a material. Fig. 5a displays the UV-Vis spectra of Eu^3+^-doped ZnO samples as the Eu^3+^ doping concentration varies. ZnO is a wide bandgap semiconductor with a direct bandgap of approximately 3.37 eV, corresponding to the ultraviolet (UV) region of the spectrum.^1,12,15^ In the absorption spectra of Z0–Z7 samples, a prominent absorption edge appears from 339.07 to 347.82 nm (see Table 2), which is attributed to the band-to-band transition, where electrons are excited from the valence band to the conduction band of the ZnO host.^1,15^ For ZnO QDs, the absorption shows a blue shift compared to bulk ZnO due to the quantum confinement effect. This phenomenon occurs when the size of the QDs becomes comparable to or smaller than the exciton Bohr radius. As a result, the bandgap energy increases (see Table 2), shifting the absorption edge to shorter wavelengths (higher energies). The magnitude of this shift depends on the size of the QDs; smaller QDs exhibit larger shifts.

**Table 2:** Abs peak, band gap, and size of ZnO : Eu^3+^ QDs

Sample	Abs peak (nm)	*E*_g_ (eV)	Size (nm)	1LO_ZnO_ (cm^−1^)	1SO_ZnO_ (cm^−1^)
Z0	339.1	3.61	8.86	573.11	493.34
Z0.5	340.6	3.57	9.76	571.35	486.89
Z1	341.4	3.53	10.72	570.64	481.76
Z3	342.6	3.49	11.46	569.83	478.65
Z5	345.3	3.46	12.04	567.38	472.65
Z7	347.8	3.45	12.20	566.89	466.93

When Eu^3+^ ions are introduced into the ZnO crystal lattice, their absorption spectra undergo significant changes. The UV-Vis spectra of Eu^3+^-doped ZnO QDs show a red shift of the absorption peaks as the Eu^3+^ concentration increases. This redshift is often attributed to lattice strain, defect formation, and the introduction of Eu^3+^ ions, which modify the local electronic structure.^26,27^ Additionally, Eu^3+^ ions can interact with oxygen vacancies, leading to changes in the defect states within the bandgap, further influencing the absorption spectrum.^27,28^ The absorption peaks become broader as the Eu^3+^ concentration increases, confirming that the incorporation of Eu^3+^ ions affects the optical transitions in the material.^27–29^ Eu^3+^ ions, which typically occupy Zn^2+^ sites in the ZnO lattice, introduce localized energy states within the bandgap of ZnO. These localized states affect the electronic transitions and optical properties of the QDs. The absorption bands associated with the intra-4f electronic transitions of Eu^3+^ ions are not visible in the 300–400 nm range of Fig. 5a. These transitions are typically much weaker than the absorption of ZnO because the intra-4f transitions of Eu^3+^ ions are parity-forbidden.^26,28,29^ The band-gap energies (*E*_g_) for both undoped ZnO and Eu^3+^-doped ZnO QDs were estimated by extrapolating the linear portion of the (*αhv*)^2^*versus* energy (*hv*) plots (Fig. 5b):^30^

6 *αhv* = *A*(*hv* − *E*_g_)^*n*^

where *A* is a constant, *hv* is the photon energy, *E*_g_ is the optical band gap, *h* is Planck's constant, *α* is the absorption coefficient, and *n* represents the index corresponding to distinct values (2, 3, 1/2, and 1/3) signifying indirect allowed, indirect forbidden, direct allowed, and direct forbidden transitions, respectively. ZnO is a semiconductor characterized by a direct band gap where *n* = 1/2. The optical band gap was determined by extrapolating the linear segment of the plot of (*αhv*)^2^*versus* (*hv*) to the energy axis. The relationship between (*αhv*)^2^ and (*hv*) for ZnO and Eu^3+^-doped ZnO QDs is illustrated in Fig. 5b. The optical band-gap of the pure ZnO QDs is estimated to be 3.61 eV. When Eu^3+^ ions were incorporated in the ZnO host, the bandgap energy of Eu^3+^-doped ZnO QDs decreases linearly with Eu^3+^ concentration from 3.57 to 3.45 eV. The reduced band gap of Eu^3+^-doped ZnO QDs compared to undoped ZnO QDs arises from a combination of factors, including defect formation, lattice distortion, Eu-induced electronic states, and a reduction in the quantum confined effect due to the size change of the QDs.^15,17,18^ Among the above reasons, the size change of ZnO QDs due to Eu^3+^ doping may be the main cause.

The size of QDs was determined based on their band gap energy (*E*_g_) using the effective mass approximation (EMA) model, which relates the size of semiconductor QDs to their band gap due to quantum confinement effects. The size of QDs was estimated using the following equation for the quantum confinement effect in a spherical particle:^26,31^7
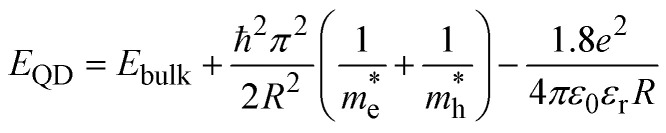
where *E*_QD_ is the band gap of the QD with radius *R*. *E*_bulk_ is the bulk band gap energy (3.37 eV for ZnO). *ℏ* is the reduced Planck's constant. 
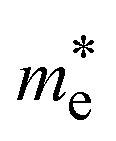
 is the effective mass of the electron in ZnO (
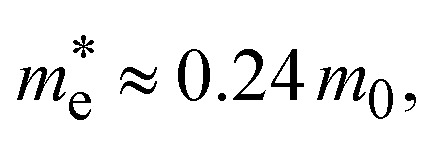
 where *m*_0_ is the free electron mass). 
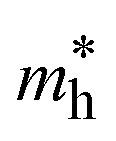
 is the effective mass of the hole in ZnO 
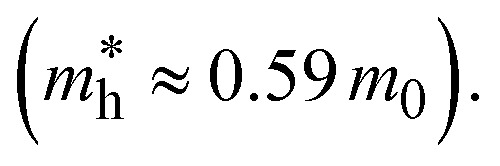
*ε*_0_ is the permittivity of free space. *ε*_r_ is the relative permittivity of ZnO (*ε*_r_ ≈ 8.5), and *e* is the elementary charge.^1,32^ The sizes of the QDs were determined and given in Table 2.

### 3.4. Photoluminescence properties

**Fig. 6:**
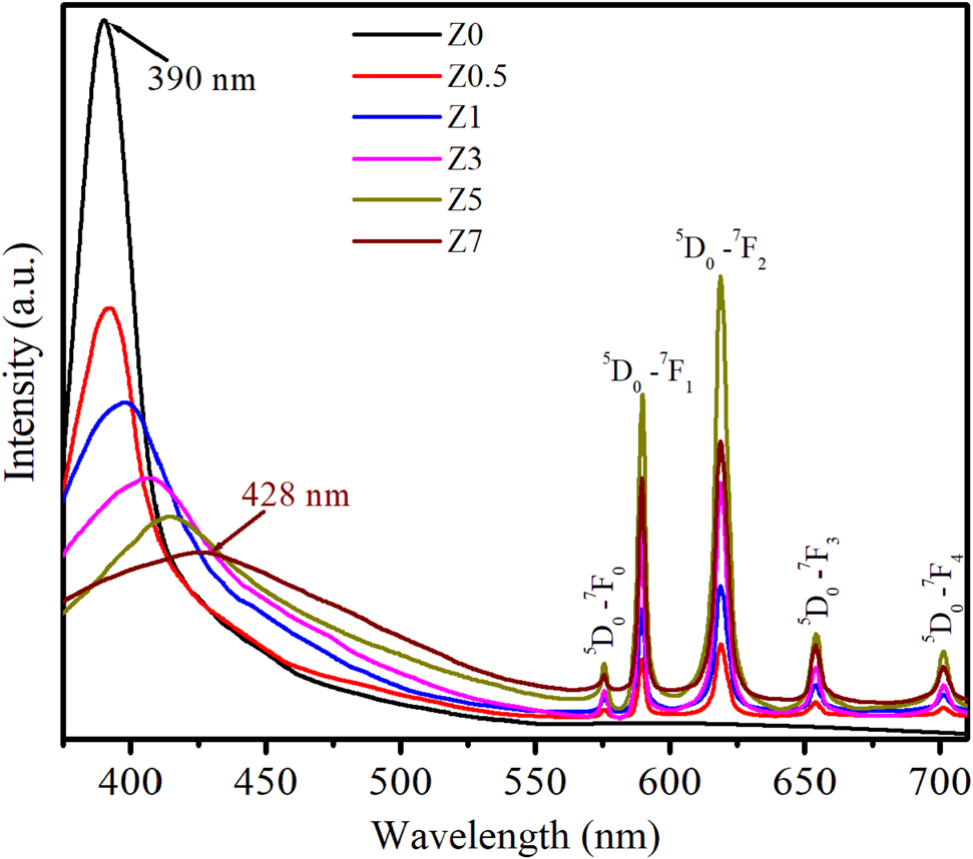
PL spectra of Eu^3+^-doped ZnO QDs with various concentrations of Eu^3+^ ions.

The photoluminescence (PL) spectra of ZnO and Eu^3+^-doped ZnO QDs under 330 nm excitation are presented in Fig. 6. The PL spectra in Fig. 6 display both the intrinsic emission of the ZnO host and the characteristic sharp emission lines of Eu^3+^ ions, corresponding to transitions between the excited ^5^D_0_ state and lower-energy ^7^F_*J*_ (*J* = 0, 1, 2, 3, 4) levels.^2,15^ The broad emission band centered around 390–428 nm, observed prominently in the undoped and doped samples, is attributed to the near-band-edge (NBE) emission of the ZnO host, which originates from excitonic recombination processes.^1,18^ This emission intensity decreased with increasing Eu^3+^ doping, suggesting that the presence of Eu^3+^ ions likely induces non-radiative processes or energy transfer mechanisms that quench the NBE emission of ZnO QDs. This quenching effect becomes apparent as the Eu^3+^ concentration increases. Fig. 6 shows that the NBE emission of the ZnO host shifts toward longer wavelengths (red-shift) with increasing Eu concentration. This red-shift is a consequence of several factors, including increased lattice stress, the formation of more defects, enhanced energy transfer between the ZnO host and the Eu^3+^ ions, and changes in QD size.^17,18^ As the Eu^3+^ concentration increases, these effects are enhanced, reducing the energy of the emitted photons, shifting the NBE emission toward longer wavelengths (lower energies).

For Eu^3+^-doped ZnO QDs, the distinct peaks between 570 nm and 710 nm are characteristic of the Eu^3+^ ion transitions (4f transitions). These transitions are less affected by the surrounding crystal field due to the shielding by the 5s and 5p electrons. The strong emissions correspond to the transitions: ^5^D_0_ → ^7^F_0_ (575 nm), ^5^D_0_ → ^7^F_1_ (589 nm), ^5^D_0_ → ^7^F_2_ (619 nm), ^5^D_0_ → ^7^F_3_ (654 nm), and ^5^D_0_ → ^7^F_4_ (701 nm).^2,17^ In these transitions, ^5^D_0_ → ^7^F_1_ is a magnetic dipole transition, which is less sensitive to crystal field symmetry. The ^5^D_0_ → ^7^F_2_ is an electric dipole transition that is sensitive to the local environment, typically stronger at low-symmetry sites.^13,14,26^

As the Eu^3+^ concentration increases (0.5–5%), the intensity of these peaks, particularly the ^5^D_0_ → ^7^F_2_ transition at 619 nm, becomes more pronounced, demonstrating an effective incorporation of Eu^3+^ into the ZnO QDs. The ^5^D_0_ → ^7^F_2_ transition, known for its sensitivity to the asymmetry of the local crystal field, becomes dominant, suggesting that Eu^3+^ ions occupy sites within the ZnO lattice that lack inversion symmetry. As seen in the PL spectra, the emission intensity of Eu^3+^-related transitions increases as the Eu^3+^ concentration increases from 0.5 to 5%. However, when the Eu^3+^ concentration reaches 7% (sample Z7), there is a noticeable decrease in the intensity of these emissions. The observed reduction in Eu^3+^ emission intensity in sample Z7 is a typical example of concentration quenching. The main contributing factors are likely: (i) cross-relaxation between closely spaced Eu^3+^ ions, (ii) energy migration to non-radiative defects, and (iii) Eu^3+^ ions aggregating together instead of being evenly distributed in the ZnO host.^2,13,26^ These mechanisms, combined with defect formation in the ZnO host, result in the reduced efficiency of radiative recombination, leading to the observed decrease in photoluminescence intensity at high Eu^3+^ concentrations.

### 3.5. Judd–Ofelt analysis

#### 3.5.1. Intensity parameters

The Judd–Ofelt theory is a powerful tool for analyzing rare-earth ions' optical properties in solid-state materials.^1,2^ It helps to estimate parameters related to the intensity of electric dipole transitions, the local ligand field around the rare-earth ions, and the radiative properties of the material.^13^ Judd–Ofelt theory calculates three intensity parameters: *Ω*_2_, *Ω*_4_, and *Ω*_6_. These parameters are related to the local environment (or ligand field) around the rare-earth ions and the probability of electric dipole transitions. *Ω*_2_ is sensitive to the asymmetry of the ligand field surrounding the rare-earth ion and provides insight into the local symmetry.^2,13^*Ω*_4_ and *Ω*_6_ provide information on the covalency of the bonding and the rigidity of the lattice around the ion.^1^ These parameters can be calculated from absorption or PL spectra by analyzing the intensities of the electronic transitions of the Eu^3+^ ions (usually from the ground state to higher excited states). In this study, the intensity parameters were determined from the PL spectra.

The emission probabilities of the transitions between different levels in Eu^3+^ ions are key to determining the intensity parameters in the JO theory. The magnetic dipole (MD) transition (^5^D_0_ → ^7^F_1_) is nearly independent of the host material. The electric dipole (ED) transitions (^5^D_0_ → ^7^F_2_, ^5^D_0_ → ^7^F_4_, and ^5^D_0_ → ^7^F_6_) are sensitive to the host environment.^2,13^ Their emission probabilities *A*_ED_ depend on the refractive index *n* of the host, the energy of the transition *ν*, and the intensity parameters *Ω*_2_, *Ω*_4_, and *Ω*_6_. These parameters represent how the local environment (or ligand field) affects the electric dipole transitions.^37,38^8
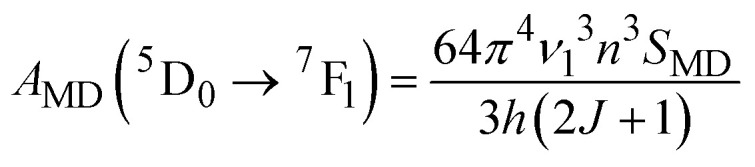
9
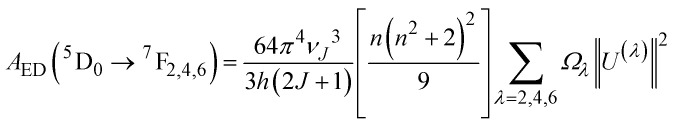
where ‖*U*^(*λ*)^‖^2^ are the squared doubly reduced matrix elements of the unit tensor operator of the rank *λ* = 2, 4, 6 for a transition |*ψJ*〉 → |*ψ*′*J*′〉. From eqn (8) and (9), one can obtain the following expression:10
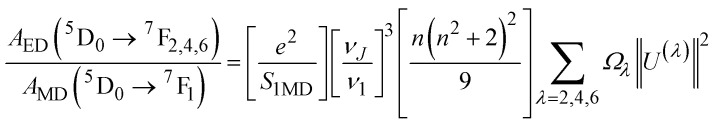
where *ν*_*J*_ (*J* = 2, 4, 6) represents the energies associated with the ^5^D_0_ → ^7^F_*J*_ (*J* = 2, 4, 6) transitions. The *A*_ED_(^5^D_0_ → ^7^F_2,4,6_)/*A*_MD_(^5^D_0_ → ^7^F_1_) ratios approximately correspond to the ratio of the integrated intensities of the ^5^D_0_ → ^7^F_*J*_ (*J* = 2, 4, 6) transitions to that of the ^5^D_0_ → ^7^F_1_ transition:^39,40^11
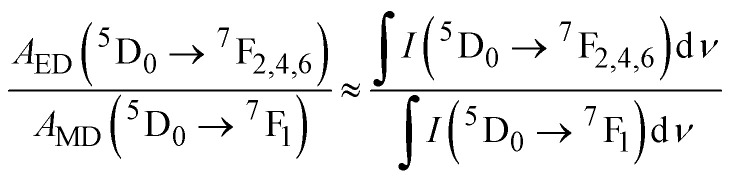


*A*_MD_ and *A*_ED_ are calculated from the integrated intensities of the PL spectrum.^41–43^ These *A*_MD_ and *A*_ED_ values are then used to calculate the JO parameters *Ω*_2_, *Ω*_4_, and *Ω*_6_. For the ^5^D_0_ → ^7^F_2_ transition: *U*^(2)^ = 0.0033, *U*^(4)^ = *U*^(6)^ = 0; for the ^5^D_0_ →^7^F_4_ transition: *U*^(2)^ = 0, *U*^(4)^ = 0.0023, *U*^(6)^ = 0; for the ^5^D_0_ → ^7^F_6_ transition: *U*^(2)^ = *U*^(4)^ = 0, *U*^(6)^ = 0.0003.

The JO parameters *Ω*_2_, *Ω*_4_, and *Ω*_6_ are determined and given by Table 3. The *Ω*_2_ values for the ZnO : Eu^3+^ QDs range from 3.005 to 3.445. These values indicate a moderate degree of asymmetry and covalency around Eu^3+^ ions in ZnO : Eu^3+^ QDs. The highest *Ω*_2_ value is seen in Z3, indicating that the Z3 sample has the most asymmetric environment among the ZnO : Eu^3+^ QDs, possibly due to an optimal balance between the doping concentration and host environment. The results in Table 3 show that the *Ω*_2_ values in Eu (0.5–7%)-doped ZnO samples are similar to those in CdS and ZnS semiconductor hosts. This indicates similar local asymmetry around Eu^3+^ ions in these II–VI semiconductor hosts. The *Ω*_4_ values in Eu-doped ZnO QDs show that the rigidity of the ZnO : Eu^3+^ environment is similar to that of ZnS and La_2_(MoO_4_)_3_ hosts, but less rigid than the CdS host. The moderate rigidity in ZnO : Eu^3+^ QDs allows for some flexibility, which may be beneficial for applications where mechanical or thermal adaptability is required. The parameter *Ω*_6_ is related to higher order interactions and is determined from the ^5^D_0_ → ^7^F_6_ transition in the wavelength range of about 725–735 nm. However, in Eu^3+^-doped materials the higher order effects are negligible, so the absence of the *Ω*_6_ parameter does not affect the analytical results.

**Table 3:** The JO intensity parameters of Eu^3+^ ion in some hosts

Sample	*Ω*_2_ (10^−20^ cm^2^)	*Ω*_4_ (10^−20^ cm^2^)	*Ω*_6_ (10^−20^ cm^2^)	Ref
Z0.5	3.236	1.009	—	This work
Z1	3.050	0.983	—	This work
Z3	3.445	1.187	—	This work
Z5	3.113	1.015	—	This work
Z7	3.005	0.971	—	This work
CdS : Eu^3+^0.5%	3.14	2.23	—	13
ZnS : Eu^3+^0.5%	3.30	1.12	—	26
La_2_(MoO_4_)_3_ : Eu^3+^	10.70	1.07	0.56	37

#### 3.5.2. Lifetime of the ^5^D_0_ → ^7^F_0_ transition and quantum efficiency of the ^5^D_0_ level

**Fig. 7:**
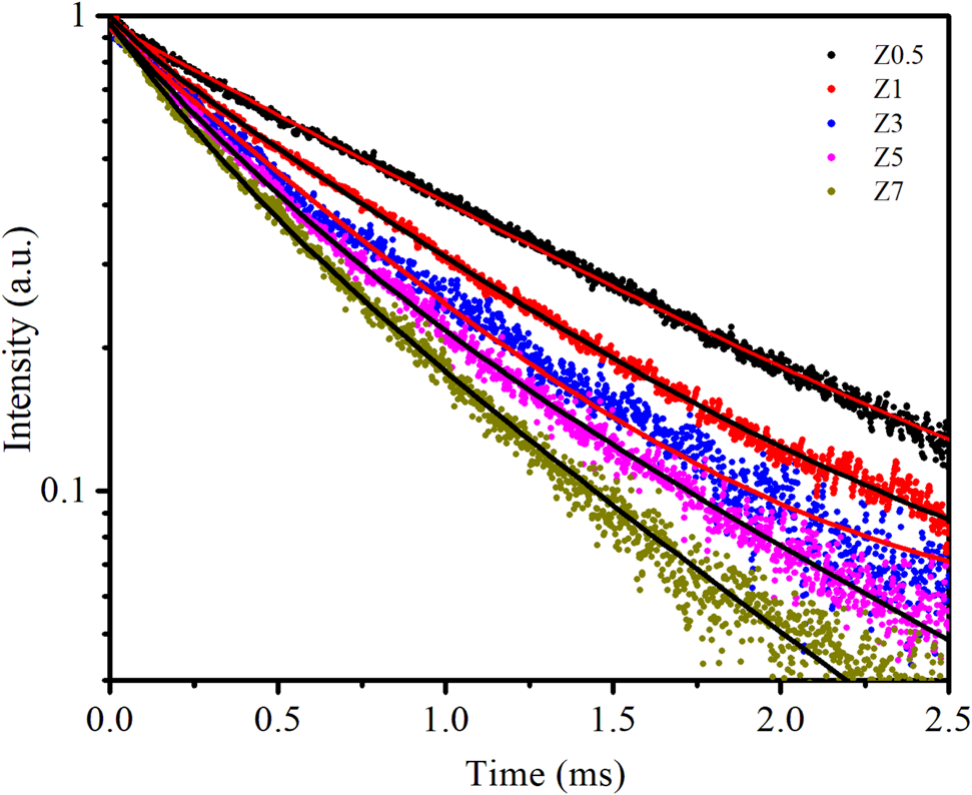
The PL decay curves of samples. The solid line is the fitted result by eqn (18).

Photoluminescence (PL) decay analysis is a crucial technique used in materials science, especially in the study of rare earth-doped semiconductors. It helps to understand the dynamic processes of excited states in materials and provides information about the electronic structure, energy transfer mechanisms, and defect states of the material.^2,13,26^Fig. 7 shows the PL decay curves of the ^5^D_0_ → ^7^F_0_ transition (619 nm) of Eu^3+^ ions in all samples under excitation at 330 nm.

The PL decay curves are fitted to a bi-exponential decay equation, which is typically written as follows:^2,13^12
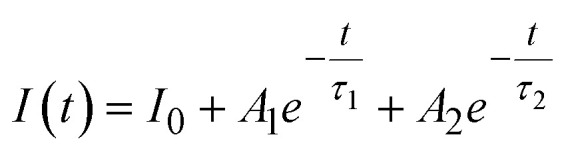
where *I*(*t*) is the PL intensity at time *t*. *I*_0_ is the initial PL intensity at *t* = 0. *A*_1_ and *A*_2_ are the pre-exponential factors corresponding to the two decay components. *τ*_1_ and *τ*_2_ are the lifetimes of the respective decay components. The bi-exponential model is used when there are two dominant recombination pathways in the sample (*e.g.*, radiative and non-radiative processes). The average lifetime 〈*τ*〉 is given by the following equation:^2,13^13
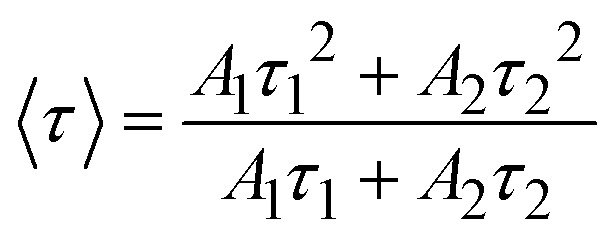


The obtained values of *A*_*i*_, *t*_*i*_, and 〈*τ*〉 by fitting are listed in Table 4. As seen in Table 4, the average lifetime 〈*τ*〉 progressively decreases from 1.11 ms in sample Z0.5 to 0.50 ms in sample Z7 as the Eu^3+^ ion concentration increased in the samples. A shorter average lifetime indicates faster non-radiative recombination or more efficient energy transfer processes, meaning that as the Eu^3+^ concentration increases, the ZnO host material more effectively transfers energy to the Eu^3+^ ions. This accelerates the decay of the PL, resulting in shorter lifetimes. The decrease in 〈*τ*〉 as Eu^3+^ concentration increases provides strong evidence of the energy transfer process from the ZnO host to the Eu^3+^ ions.

**Table 4:** Fitting time constants of PL decay kinetics of Eu-doped ZnO QDs

Sample	*A*_1_ (%)	*t*_1_ (ms)	*A*_2_ (%)	*t*_2_ (ms)	<*t*> (ms)
	±0.42	±0.015	±0.58	±0.012	±0.014
Z0.5	64.53	1.24	35.47	0.57	1.11
Z1	54.71	0.97	45.29	0.49	0.83
Z3	59.34	0.77	40.66	0.44	0.68
Z5	61.25	0.66	38.75	0.35	0.58
Z7	67.33	0.55	32.67	0.34	0.50

The quantum efficiency of the ^5^D_0_ level of Eu^3+^ is a crucial parameter in evaluating the performance of Eu^3+^-doped materials, particularly for applications in luminescent devices. This level corresponds to the lowest excited state of Eu^3+^, from which radiative transitions occur to the ground state, primarily involving the ^7^F_*J*_ (*J* = 0–4) levels, resulting in the characteristic red luminescence.^37,38^Fig. 7 shows the decay curves of the ^5^D_0_ level of the Eu^3+^ ion in Eu-doped ZnO QDs. The experimental lifetime (*τ*_exp_) was determined by fitting the PL decay curves using a bi-exponential function. The lifetimes of samples are presented in Table 5. The quantum efficiency (*η*) of the ^5^D_0_ level and the energy transfer probability (*W*_ET_) from this level are determined using the following formulas:^13,42^14
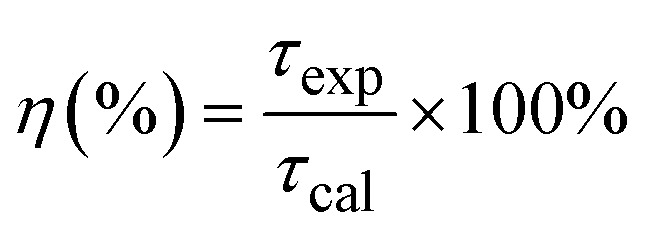
15
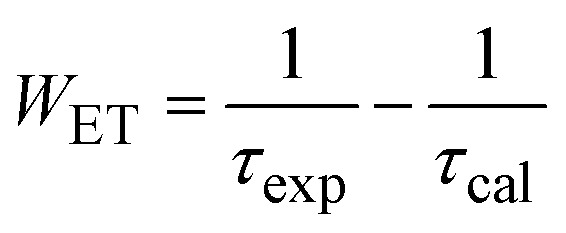
where *τ*_exp_ is the experimental lifetime. *τ*_cal_ is the lifetime, which is calculated by JO theory. This formula expresses the ratio of the actual photon emission to the maximum possible photon emission if all excitations result in radiative transitions.

**Table 5:** The calculated lifetime (*τ*_cal_, μs), experimental lifetime (*τ*_exp_, μs), quantum efficiency (*η*, %), and energy transfer probability (*W*_ET_, s^−1^) from the ^5^D_0_ level

Sample	*τ*_cal_	*τ*_exp_	*η*	*W*_ET_
Z0.5	1333	1110	83.27	149.02
Z1	1388	830	59.80	480.18
Z3	1259	680	54.01	676.31
Z5	1366	580	42.46	992.07
Z7	1402	500	35.66	1286.73

The results presented in Table 5 demonstrate a clear trend of decreasing quantum efficiency and increasing energy transfer rate with rising concentrations of Eu^3+^ ions in the Eu-doped ZnO QDs. As the concentration of Eu^3+^ increases, the average distance between Eu^3+^ ions decreases, intensifying the interaction between them. This enhanced interaction facilitates a more efficient energy transfer between Eu^3+^ ions. The observed reduction in the lifetime of the ^5^D_0_ level in Eu^3+^ can be attributed to the migration of excitation energy between Eu^3+^ ions.^13,26^ During this process, the Eu^3+^ ion in the ^5^D_0_ state relaxes to the ^7^F_0_ level by transferring its energy to a neighboring Eu^3+^ ion, exciting it from the ^7^F_0_ to the ^5^D_0_ state.^38^ This results in the migration of excitation energy through multiple Eu^3+^ ions before photon emission occurs. In addition to energy transfer between Eu^3+^ ions, the presence of defects in the material acts as an additional mechanism for luminescence quenching.^39,40^ These defects act as acceptor centers, which are capable of trapping excitation energy from nearby Eu^3+^ ions if they are close enough. Once trapped, the defects may relax back to their ground state by emitting multiphonon or infrared radiation, leading to non-radiative decay and reducing the overall luminescence of the Eu^3+^ ions. As the concentration of Eu^3+^ increases, the rate of energy trapping by defect centers also rises, resulting in a quenching effect. This is reflected in the reduction of the fluorescence lifetime and fluorescence intensity when the Eu^3+^ concentration exceeds 5%.

### 3.6. Energy transfer from the ZnO host to Eu^3+^ ions

**Fig. 8:**
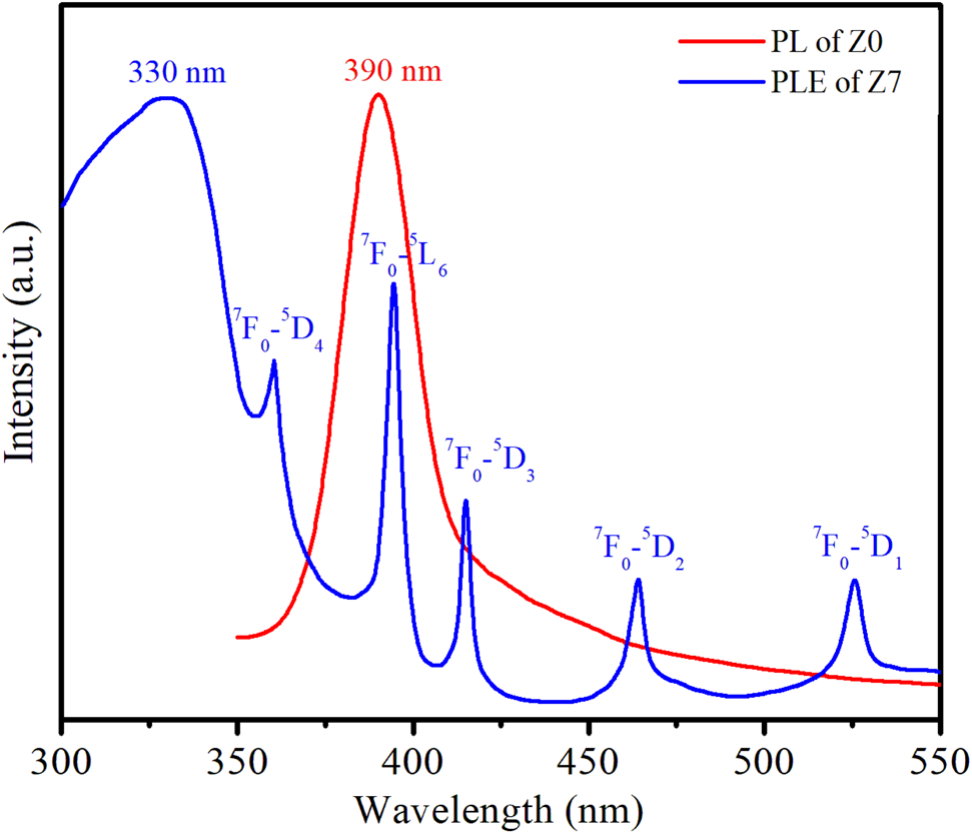
PL spectrum of the Z0 sample and PLE spectrum of the Z7 sample.

Fig. 8 shows the PL spectrum of the Z0 sample and the photoluminescence excitation (PLE) spectrum of the Z7 sample. To obtain the PLE spectrum, the emission wavelength was fixed at 619 nm, corresponding to the ^5^D_0_ → ^7^F_2_ transition of Eu^3+^ ions.

**Fig. 9:**
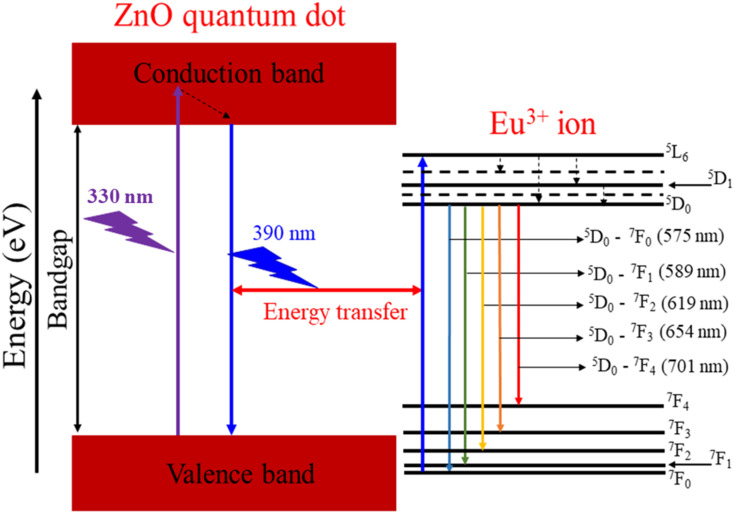
Schematic energy level diagram and ET process from the ZnO host to Eu ions.

The PLE spectrum of Z7 reveals six distinct peaks. The broad peak centered at 330 nm is related to the absorption of the ZnO host. The next five narrow peaks are transitions between the ground state ^7^F_0_ of Eu^3+^ and various excited states (^5^D_4_, ^5^L_6_, ^5^D_3_, ^5^D_2_, and ^5^D_1_) of the Eu^3+^ ions at wavelengths of 360, 394, 415, 464, and 525 nm, respectively. A strong peak in the PL spectrum of the Z0 sample is observed around 390 nm, which corresponds to the near-band-edge emission of ZnO QDs.^1,15^ This emission is characteristic of excitonic recombination in ZnO QDs. The ^7^F_0_ → ^5^L_6_ excitation peak of Eu^3+^ ions is entirely situated within the emission region of the ZnO QDs. This alignment indicates efficient energy transfer from the ZnO host material to the Eu^3+^ ions.^17,18^ The ZnO host absorbs energy and subsequently transfers it to the Eu^3+^ ions, facilitating their excitation. The energy transfer process from the ZnO host to the Eu^3+^ ions is depicted schematically in Fig. 9. The energy absorbed by the ZnO host is transferred to the Eu^3+^ ions, resulting in their characteristic red luminescence. This process demonstrates the efficient energy transfer between the ZnO host and the Eu^3+^ dopants, enhancing the luminescent properties of the material.^15–17^

Fig. 9 illustrates the energy level diagram and energy transfer process from the ZnO QDs to Eu^3+^ ions. This diagram helps explain the PL and energy transfer mechanism between the ZnO host material and the Eu^3+^ dopant ions. When the ZnO QDs are excited by UV light (at 330 nm), electrons in the valence band of ZnO are excited into the conduction band, creating electron–hole pairs. After being excited, the electrons return to the valence band and emit a wavelength of 390 nm. Part of this radiation energy is transferred to the Eu^3+^ ions and excites them to move from ^7^F_0_ to ^5^L_6_. From the ^5^L_6_ state, the electrons relax non-radiatively to the ^5^D_0_ state. Then, the Eu^3+^ ions from the ^5^D_0_ state return to the ^7^F_*J*_ (*J* = 0–4) states and emit photons at specific wavelengths of 575, 589, 619, 654, and 701 nm corresponding to the ^5^D_0_–^7^F_0_, ^5^D_0_–^7^F_1_, ^5^D_0_–^7^F_2_, ^5^D_0_–^7^F_3_, and ^5^D_0_–^7^F_4_ transitions. It should be noted that the excitation wavelength of 330 nm used coincides with the excitation peak of the ZnO host and does not coincide with any other excitation peak of Eu^3+^ ions. The wavelength of 330 nm is not suitable for the excitation of Eu^3+^ ions.

The efficiency of energy transfer (*η*_ET_) from a ZnO host to Eu^3+^ ions is an important parameter that determines how effectively the ZnO host transfers its absorbed energy to the Eu^3+^ ions, leading to characteristic Eu^3+^ emission. The *η*_ET_ can be calculated from the change in the PL intensity of the ZnO host using the formula:^13^16
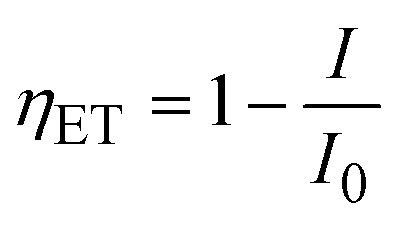
where *I* and *I*_0_ represent the integrated luminescence intensities of ZnO in the presence and absence of Eu^3+^ ions, respectively; the *η*_ET_ was calculated and presented in Table 6. The data show that as the concentration of Eu^3+^ ions increases from 0.5 to 7%, the *η*_ET_ rises from 40.9% to 75.4%. This shows a clear trend where higher Eu^3+^doping levels in ZnO QDs lead to more efficient energy transfer from the ZnO host to the Eu^3+^ ions. The increased *η*_ET_ can be explained by the fact that with higher Eu^3+^ concentrations, there are more available Eu^3+^ ions to accept the energy from the ZnO host. This enhances the non-radiative energy transfer process from ZnO to Eu^3+^ ions, resulting in more efficient excitation of Eu^3+^.

**Table 6:** The luminescence ratio (*R* = *I*(^5^D_0_–^7^F_2_)/*I*(^5^D_0_–^7^F_1_)), *η*_ET_ from ZnO QDs to Eu^3+^ ions, chromaticity coordinates (*x*, *y*) and the CCT for Eu^3+^-doped ZnO QDs

Sample	*x*	*y*	CCT (K)	*R*	*η*_ET_ (%)
Z0	0.164	0.071	1682.06	—	—
Z0.5	0.229	0.141	3010.63	1.24	40.9
Z1	0.244	0.149	3209.27	1.20	54.6
Z3	0.249	0.154	3791.77	1.23	64.73
Z5	0.299	0.211	21957.22	1.36	70.48
Z7	0.276	0.218	30410.34	1.18	75.4

**Fig. 10:**
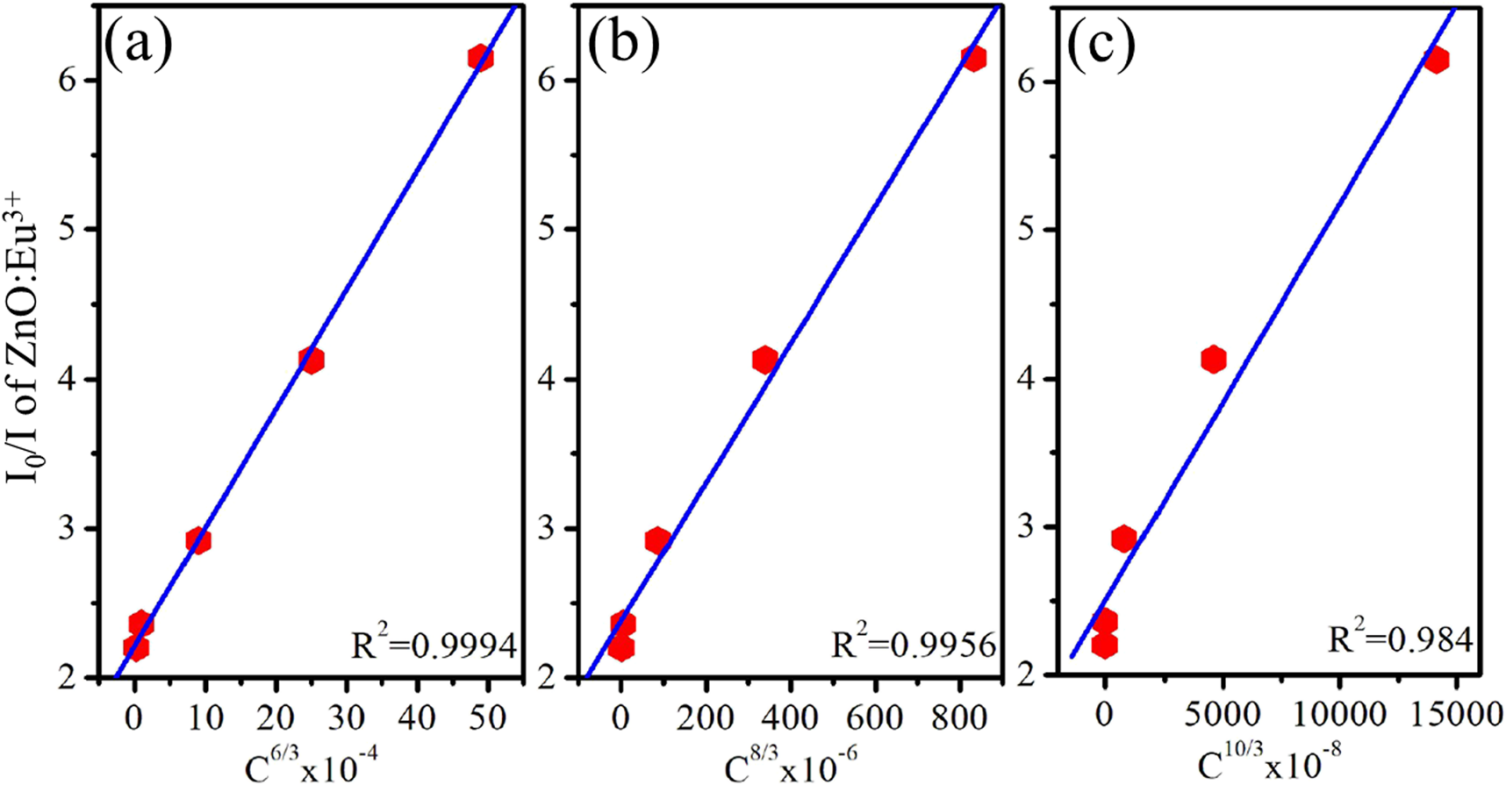
Dependence of the *I*_0_/*I* ratio on *C*^*α*/3^: (a) *α* = 6, (b) *α* = 8, (c) *α* = 10.

Reisfeld's approximation is a useful theoretical tool for analyzing the energy transfer (ET) process between a host material and dopant ions. Reisfeld's approximation provides a mathematical equation for determining the energy transfer efficiency (*η*_ET_) based on the concentration of the acceptor ions (Eu^3+^) and the distance-dependent interaction between the donor (ZnO) and acceptor (Eu^3+^). The general form of that relationship follows the equation:^13,26^17
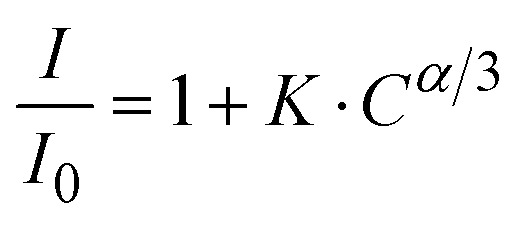
where *K* is a constant that depends on the interaction strength and other system parameters. *C* is the concentration of the Eu^3+^ ions. *α* is an exponent that characterizes the type of interaction mechanism, with typical values related to the distance dependence of the energy transfer efficiency. *α* takes values of 6, 8, and 10 corresponding to dipole–dipole (DD), dipole–quadrupole (DQ), and quadrupole–quadrupole (QQ) interactions, respectively.^13^ The plot shows a best-fit linear relationship with *α* = 6 (*R*^2^ = 0.9994) (Fig. 10). This suggests that dipole–dipole interaction is the main mechanism governing the energy transfer from the ZnO host to the Eu^3+^ ions. The dominant dipole–dipole interaction for energy transfer from a semiconductor host to Eu^3+^ ions has been observed in various host materials.^2,13^ Hosts such as ZnS, TiO_2_, CdS, and CdSe have been shown to facilitate efficient energy transfer to Eu^3+^ ions.^2,13,44,45^ This interaction mechanism is important in the development of optoelectronic devices, LEDs, and phosphors, where energy transfer from the host to the Eu^3+^ ions is crucial to achieve high luminescence efficiency.

### 3.7. CIE color coordinates

**Fig. 11:**
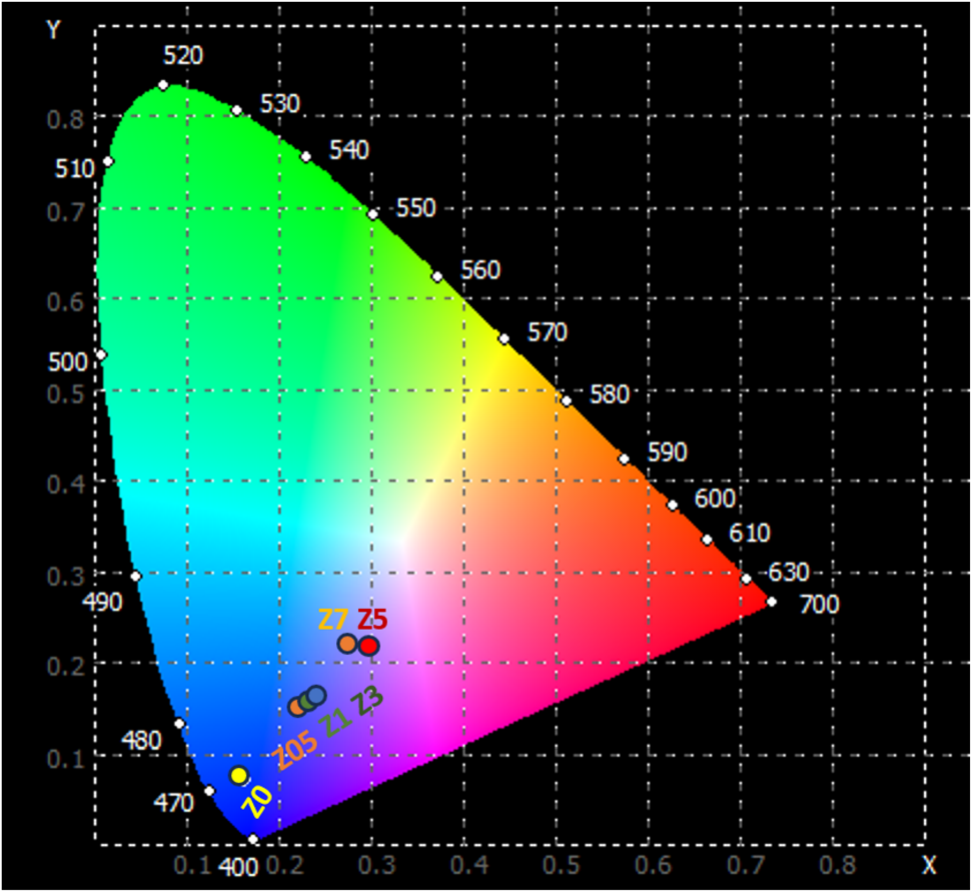
CIE of samples using the emission spectra upon excitation by 330 nm.

The emission characteristics of the material are represented in the CIE 1931 color space, a system used to describe colors based on human vision. The (*x*, *y*) chromaticity coordinates of the ZnO : Eu^3+^ QD luminescence are plotted on the CIE 1931 diagram and shown in Fig. 11. The correlated color temperature (CCT) for the samples can be determined using the McCamy approximation:^46,47^

18 CCT = −449*n*^3^ + 3525*n*^2^ − 6823*n* + 5520.33

where the *n* factor is calculated by the expression: *n* = (*x* − *x*_e_)/(*y* − *y*_e_) with *x*_e_ = 0.332 and *y*_e_ = 0.186. The CCT of samples is displayed in Table 6. For the Z0 sample, which represents undoped ZnO QDs, chromaticity coordinates (*x*, *y*) = (0.164, 0.071). The location of these coordinates suggests that the undoped ZnO QDs emit primarily in the ultraviolet to violet region. The CCT of 1682 K for ZnO QDs indicates that the light emitted by these QDs has a relatively low color temperature. This CCT value corresponds to warmer light, often resembling the light from candlelight or early sunrise.

The Z0.5, Z1, and Z3 samples exhibit progressively higher *x* and *y* coordinates, indicating a shift towards the green and blue regions of the chromaticity diagram. Their CCT values of 3010.63, 3209.27, and 3791.77 K, respectively, indicate their emission in the cooler, more neutral white light region. The Z5 and Z7 samples exhibit very high CCTs, indicating that they emit light in the cooler region of the spectrum (from blue to ultraviolet). These extremely high CCT values are similar to light emitted from natural sources such as a clear blue sky or daylight at high altitudes. For the human eye, the light from Z5 and Z7 appears as a vivid, pure blue. This is the type of light typically associated with strong artificial light, such as high-power blue LEDs or cool-tone backlighting in electronic devices. These optical properties make them suitable for many technological and biomedical applications, especially where strong blue or ultraviolet light is required.

## 4. Conclusion

Eu^3+^-doped ZnO QDs with concentrations varying from 0 to 7% were successfully fabricated by a wet chemical method. The synthesis, as well as structural, vibrational, and optical properties, and energy transfer mechanism from the ZnO host to Eu^3+^ ions were studied and explained in detail. XRD demonstrated that the Eu-doped ZnO QDs had a wurtzite structure (ICDD file no: 36-1451) with the crystallite sizes of about 7–8 nm. The valence state of the Eu ion (Eu^3+^) was verified through the characteristic Eu 3d peaks in XPS spectra. The 1LO_ZnO_ RS peak red-shifted from 573.11 to 566.89 cm^−1^ as the Eu concentration increased from 0–7%, indicating the strong binding between Eu^3+^ ions and the ZnO host. The PL spectra of samples display both the intrinsic emission of the ZnO host and the characteristic sharp emission lines of Eu^3+^ ions, corresponding to transitions ^5^D_0_ → ^7^F_0_ (575 nm), ^5^D_0_ → ^7^F_1_ (589 nm), ^5^D_0_ → ^7^F_2_ (619 nm), ^5^D_0_ → ^7^F_3_ (654 nm), and ^5^D_0_ → ^7^F_4_ (701 nm). The important parameters such as *Ω*_2_ and *Ω*_4_, branching ratios, and radiative lifetimes were determined using the Judd–Ofelt (JO) theory. The obtained *Ω*_2_ and *Ω*_4_ values indicate that the degree of asymmetry, covalent bonding, and moderate hardness around the Eu^3+^ ions in ZnO : Eu^3+^ QDs allow for flexible control of the mechanical or thermal adaptability of the material. The energy transfer process between the ZnO host and the Eu^3+^ dopant was shown to be highly efficient under the excitation wavelength of 330 nm, enhancing the emission of Eu^3+^ ions. Based on Reisfeld's approximation, the D–D interaction was determined to be the main mechanism for energy transfer from the ZnO host and the Eu^3+^ dopant. The energy transfer efficiency from the ZnO host and the Eu^3+^ dopant increases from 40.9 to 75.4% when the Eu ion concentration increases from 0.5 to 7%. These results highlight the potential of Eu^3+^-doped ZnO QDs for applications in photonic devices, light-emitting diodes (LEDs), and bio-imaging due to their tunable optical properties and efficient energy transfer mechanisms.

## Data availability

“The data supporting this study's findings are available on request from the corresponding author, Nguyen Xuan Ca, email: canx@tnus.edu.vn.”

## Conflicts of interest

There are no conflicts to declare.
